# Molecular Mechanisms behind Safranal’s Toxicity to HepG2 Cells from Dual Omics

**DOI:** 10.3390/antiox11061125

**Published:** 2022-06-07

**Authors:** David Roy Nelson, Ala’a Al Hrout, Amnah Salem Alzahmi, Amphun Chaiboonchoe, Amr Amin, Kourosh Salehi-Ashtiani

**Affiliations:** 1 Center for Genomics and Systems Biology, New York University Abu Dhabi, Abu Dhabi P.O. Box 129188, United Arab Emirates; drn2@nyu.edu (D.R.N.); amnah.alzahmi@nyu.edu (A.S.A.); 2 Biology Department, United Arab Emirates University, Al-Ain P.O. Box 15551, United Arab Emirates; 200935311@uaeu.ac.ae; 3 Institute of Experimental Immunology, University of Zürich, CH-8006 Zürich, Switzerland; 4 Division of Science and Math, New York University Abu Dhabi, Abu Dhabi P.O. Box 129188, United Arab Emirates; amphun.cha@mahidol.ac.th; 5 Siriraj Center of Research Excellence for Precision Medicine and Systems Pharmacology (SiCORE-PM&SP), Department of Pharmacology, Faculty of Medicine, Siriraj Hospital, Mahidol University, Bangkok 10400, Thailand; 6 The College, The University of Chicago, Chicago, IL 60637, USA

**Keywords:** cancer, saffron, safranal, natural products, DNA damage, hepatocellular carcinoma, hypoxanthine

## Abstract

The spice saffron (*Crocus sativus*) has anticancer activity in several human tissues, but the molecular mechanisms underlying its potential therapeutic effects are poorly understood. We investigated the impact of safranal, a small molecule secondary metabolite from saffron, on the HCC cell line HepG2 using untargeted metabolomics (HPLC–MS) and transcriptomics (RNAseq). Increases in glutathione disulfide and other biomarkers for oxidative damage contrasted with lower levels of the antioxidants biliverdin IX (139-fold decrease, *p* = 5.3 × 10^5^), the ubiquinol precursor 3-4-dihydroxy-5-all-trans-decaprenylbenzoate (3-fold decrease, *p* = 1.9 × 10^−5^), and resolvin E1 (−3282-fold decrease, *p* = 4^5^), which indicates sensitization to reactive oxygen species. We observed a significant increase in intracellular hypoxanthine (538-fold increase, *p* = 7.7 × 10^−6^) that may be primarily responsible for oxidative damage in HCC after safranal treatment. The accumulation of free fatty acids and other biomarkers, such as S-methyl-5′-thioadenosine, are consistent with safranal-induced mitochondrial de-uncoupling and explains the sharp increase in hypoxanthine we observed. Overall, the dual omics datasets describe routes to widespread protein destabilization and DNA damage from safranal-induced oxidative stress in HCC cells.

## 1. Introduction

Hepatocellular carcinoma (HCC) is the third-most leading cause of cancer death worldwide [1]. Risk factors for HCC include chronic infection with hepatitis B virus (HBV), hepatitis C virus (HCV), alcoholic liver disease, and nonalcoholic steatohepatitis (NASH). Aflatoxin-contaminated food, diabetes, obesity, certain hereditary conditions such as hemochromatosis, and various metabolic disorders increase the risk of acquiring HCC [2]. Treatment of HCC differs among patients according to their background and the tumor progression. Current therapies include transarterial embolization (TAE) (embolic particles without chemotherapy) or TACE (chemotherapeutic drugs and embolic particles), surgical resection, and liver transplantation [3]. Further research is essential to develop targeted novel therapies that address this lethal cancer and improve patient care.

**Figure 1:**
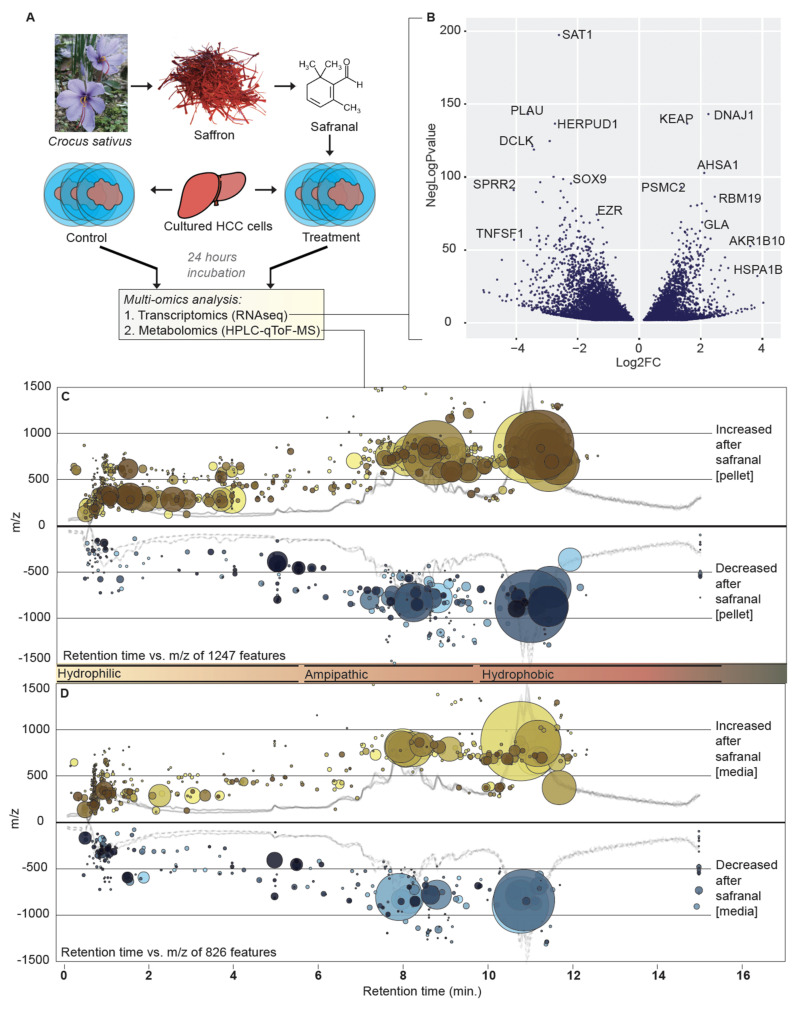
Overview of experiment and omics datasets. (**A**) Safranal is an active small molecule compound in saffron derived from the pistils of *Crocus sativus.* We performed RNAseq and HPLC–MS on extracts from safranal-treated and non-treated HCC cells. (**B**) Volcano plot showing transcript expression changes (*x*-axis) and negative log *p*-values (*y*-axis). (**C**,**D**) Mass-to-charge ratios (*m/z; y*-axis), retention time (*x*-axis), and fold-change (bubble size) of metabolites in response to safranal treatment (*p* < 0.001 for all shown features) in (**C**) cell pellet extracts or (**D**) conditioned media extracts. Yellow bubbles indicate increased, and blue indicates decreased compounds in HCC pellet extracts after safranal treatment.

Natural products have been used to produce effective anticancer drugs and adjuvant chemotherapy drugs. The induction of autophagic apoptosis by naturally sourced antitumor chemicals has been recently investigated [4]. Here we focused on the compound safranal. Safranal is produced by *Cuminum cyminum* (cumin seed), *Aspalathus linearis* (rooibos), *Centaurea sibthorpii*, *Calycopteris floribunda* (ukshi), *Citrus limon* (lemon), *Erodium cicutarium* (common stork’s-bill or pinweed), *Sambucus nigra* (elderberry), and the cyanobacterium *Microcystis*. The highest natural quantities of safranal can be found in saffron, the dried dark-red stigma of *Crocus sativus* (see Figure 1). Safranal is the most abundant molecule in saffron essential oil (~70%) [5]. Compounds in the spice saffron, including safranal, have antitumor effects in several tissues [6,7,8,9]. For example, safranal has anti-cancer activity in human colorectal cancer cells [8], human lung carcinomas [10], and neuroblastomas [11]. However, the metabolomic response of hepatocellular carcinoma cells (HCC) to endogenous saffron compounds, including safranal, has not yet been studied. Recent advances in metabolomic and transcriptomic technologies have accelerated dual omics analyses; in conjunction with advances in bioinformatics software, such as Mummichog (http://mummichog.org/, accessed on 20 March 2021), dysregulated metabolic reactions in model systems can be predicted with greater confidence. A dual omics approach to study safranal’s effect on a liver cancer model would shed insight into its apoptosis induction mechanism.

We previously reported safranal-induced apoptosis in HCC cells using a HepG2 cell line [12]. The HepG2 line was chosen due to its widespread use in liver cancer research as an excellent model for HCC progression. Our extensive analyses into the mechanism behind safranal’s anti-cancer mechanism were carried out using transcriptomics, cell cycle, and immunoblot analyses. We focused on critical regulators of the cell cycle, DNA damage repair, apoptosis, and effects on intrinsic and extrinsic initiator caspases and their implication in endoplasmic reticulum (ER) stress. Our work showed that safranal activates apoptosis in HCC, although further work was needed to detail the complex response to safranal and its anti-proliferative effects. We previously showed that safranal caused extensive DNA damage and elicited an unfolding response, although upstream mechanisms underlying safranal’s apoptosis induction were unclear. Dual omics approaches, such as that presented in this study, offer an orthogonal route to validate potential system-wide metabolic dysregulation occurring upon safranal treatment.

## 2. Materials and Methods

### 2.1. Cell Growth

Liver carcinoma cells (HEP-G2) were obtained from ATCC (HB-8065) and cultured in RPMI 1640 medium (Cytiva, Marlborough, MA, USA) with 10% fetal bovine serum (Sigma Aldrich, St. Louis, MO, USA), 1% 100 U/mL penicillin, and 100 µg/mL streptomycin (Sigma Aldrich, St. Louis, MO, USA) at 37 °C and 5% CO_2_. Cells were sub-cultured every 3–5 days using trypsin 0.25% (Cytiva, Marlborough, MA, USA). Biological triplicates for non-treated (vehicle only (dimethyl sulfoxide (DMSO; Sigma Aldrich, St. Louis, MO, USA))) and safranal-treated underwent viability assays, then partitioning for RNA and metabolite extractions and downstream transcriptomic and metabolomic analyses. Apoptosis was verified by a caspase cleavage assay as in ref. [12]; briefly, a Caspase-Glo^®^ 3/7 Assay kit (Promega, Madison, WI, USA) was used to monitor caspase-3 and 7 activities after safranal treatment.

### 2.2. RNA Extraction and Sequencing

HEP-G2 cells were treated with 500 uM safranal for 24 h. After 24 h, cells were centrifuged and frozen in liquid nitrogen. Cells were thawed at room temperature for 15 min, centrifuged for 15 min at 4000 RPM, and the supernatant was discarded. Pellets were resuspended in 500 μL 1x dPBS. Cells were then centrifuged at 10,000 RPM for 10 min, and cell pellets were used for RNA extraction following the Qiagen (Hilden, Germany) RNAeasy Plus Mini kit protocol.

Sequencing read analyses, including adapter content, duplication levels, kmer profiles, per-base N content, per-base quality, per base sequence content.png, per sequence G + C% content, per sequence quality, per tile quality, sequence length distribution, and FASTQC (https://www.bioinformatics.babraham.ac.uk/projects/fastqc/, accessed 23 May 2020) reports are in Data S1. Paired-end sequencing reads are hosted at the SRA database in NCBI with the BioProject accession PRJNA682690.

### 2.3. Metabolite Extraction

Cells were treated with 500 uM safranal for 24 h. After 24 h, cells were centrifuged and both cells and conditioned media were frozen in liquid nitrogen. Cell pellets and conditioned media were thawed in 5 mL methanol (Sigma-Aldrich, Darmstadt, Germany) and sonicated for 30 min with a 296 W sonicator bath (Wiseclean; Witeg, Schönwalde-Glien, Germany) followed by vortexing. Cell extracts were filtered through 0.2 uM filters (Merck Millipore, Darmstadt, Germany) and stored in color-protected amber glass HPLC vials (Agilent, Santa Clara, CA, USA) at 4 °C until column loading.

### 2.4. LC/MS-QToF

All metabolites were dissolved in methanol to achieve separations of biomolecules on a reverse-phase C18 HPLC column. All samples were run in biological triplicates, with results from technical triplicates averaged for XCMS inputs. Eluted molecules went to an Agilent LC-MS QToF 6538 with accurate mass profiling with lock mass compounds (error < 2 ppm, Agilent, Santa Clara, CA, USA). Compounds were eluted from a reverse-phase C18 column using a semi-linear isopropanol gradient as in ref. [13], which was adapted from a shotgun lipidomics protocol [14] modified to capture a wide range of intracellular metabolites inclusive of high-molecular-weight, hydrophobic compounds. Briefly, individual components in the extracts were separated in a semi-linear hydrophobicity gradient using acetonitrile, ammonium formate, and isopropanol. The separation protocol was designed to send a wide range of intact biomolecules to a quadrupole time-of-flight mass spectrometer (qTOF-MS, Agilent, Santa Clara, CA, USA). This elution scheme was designed to elute small hydrophilic molecules first and gradually induce mobility in more massive, hydrophobic molecules such as phospholipids then triacylglycerols over 14-min runs. We achieved the separation of hydrophilic and hydrophobic compounds at the initial and final HPLC column elution endpoints using this extraction procedure and solvent strategy (Figure 1).

### 2.5. Computational, Quantitative, and Statistical Analyses

Major peak clusters correspond with hydrophilic, mesophilic, and hydrophobic compounds in cell extracts. Molecular features detected by the MS were extracted for downstream untargeted metabolomics and multi-omics strategies.

Mass-to-charge molecular features were extracted with the MassHunter software (Agilent, Santa Clara, CA, USA) and uploaded to the XCMS and METLIN database for compound assignment [15,16,17,18,19]. All significant molecular features were extracted and used as queries in the XCMS software suite [15,16,17,18,19].

Overall, 2484 compounds in the conditioned media and 5550 in the pellets of safranal-treated HCC cells had significantly different accumulation after 24 h (FDR-corrected *p*-value < 0.05; Figure 1 shows different metabolites at *q* < 0.001). HPLC column retention time correction was performed in XCMS with the adjustRtime method to align chromatograms [15,16,17,18,19]. The ‘peakGroups’ method was used for retention time correction from alignments of peak groups present in most/all samples. The ‘adjustRtime-peakGroups’ method facilitates accurate comparisons between HPLC runs.

### 2.6. Differential Gene Expression Analysis

Volcano plots, DEGs, and counts were generated using RNA-seq 2G (http://52.90.192.24:3838/rnaseq2g/, accessed 20 April 2021). Significantly differentially expressed genes were identified using the cutoff values of False Discovery Rate (FDR) < 0.05 in DeSeq2 [20]. Venn diagrams were constructed using InteractiVenn (http://www.interactivenn.net, accessed 1 April 2021).

### 2.7. Pathway Analysis

DEGs were analyzed through Ingenuity Pathway Analysis (IPA) (QIAGEN Inc., https://www.qiagenbioinformatics.com/products/ingenuitypathway-analysis, accessed 18 April 2021 [21]). The cutoff value for an FDR was set to 0.01. Core analysis was performed with default settings, and canonical pathways and associated toxicity functions were reviewed. Forty-five genes that were associated with HCC were re-analyzed (core analysis) for further toxicity function association. Transcriptomic data were used to retrieve ECs from PFAMs [22], and CAS numbers were obtained from metabolite assignments in METLIN (https://metlin.scripps.edu, accessed 4 May 2021). These ECs were uploaded to iPATH3 (https://pathways.embl.de/, accessed 29 May 2021) and used to generate the overlap pathways for dual omic comparisons.

## 3. Results

### 3.1. Dual Omics Systems Biology Resolves the HCC Safranal Response

We investigated the impact of safranal on HCC cells (HEP-G2) in vitro from metabolomic and transcriptomic perspectives by combining evidence from both approaches to understand the complex mechanisms underlying the HCC apoptotic safranal response. HCC cells from safranal-treated and control culture experiments were partitioned for transcriptomics (RNAseq) and metabolomics (HPLC–MS) (*n* = 3 biological replicates for each group and in each experiment). Then, we compared control and safranal-treated HCC cells using extracted metabolites and RNA for downstream metabolomic and transcriptomic analyses.

The RNAseq experiments were performed to confirm the reproducibility of our previous work and to provide a new dataset suitable for dual omics comparisons. Transcriptomic observations supported and expanded upon our previous work [12]. As safranal has been found to promote protein destabilization [8,23], we evaluated the differential expression of genes involved in the unfolded protein response in our treated samples. DNAJ1 [24,25,26] (fold-change = 4.77, *p* = 1.04 × 10^−139^) and AHSA1 (Activator Of HSP90 ATPase Activity 1; fold-change = 4.35, *p* = 7.55 × 10^−100^) [27,28,29,30] were highly up-regulated after safranal treatment. Their upregulation suggests an elevated unfolded protein response after safranal treatment. Additionally, safranal inhibited HeLa cell viability by interfering with microtubule formation [31]. PSMC2 (ENSG00000161057), a component of the 26S proteasome, was significantly upregulated after safranal treatment (fold-change = 2.96, *p* = 3.68 × 10^−90^). PSMC2 is a multicatalytic proteinase complex whose role is to destroy unfolded proteins before they cause an intracellular catastrophe. Together with DNAJ1 and AHSA1, PSMC2 upregulation outlines a coordinated unfolded protein response after safranal treatment in HCC cells.

Overall increases in identifiable biomolecules in media extracts indicated higher cell lysis levels after treatment. Hydrophilic molecules in the conditioned media and pellets after safranal treatment were significantly increased (retention time < 5 min, see Figure 1). A significant decrease in large, hydrophobic molecules, such as tri- and di-glycerides (TAGs and DAGs, respectively) in the media of the safranal-treated cells was observed (Figure 1, see also Table S2).

Cell pellets did not show any significant difference in total TAGs after safranal treatment; however, hydrophilic compounds were significantly increased in these fractions (Figure 1). We saw lower levels of degradation products in several pathways, including sorbitol degradation I (5-fold decrease, *p* = 3.1 × 10^−5^), heme degradation (3281-fold decrease, *p* = 5.3 × 10^−5^), thymine degradation (3.7-fold decrease, *p* = 1.3 × 10^−5^), tryptophan degradation (4.3-fold increase, *p* = 5 × 10^−5),^ and hydroxyproline degradation (3.7-fold change, *p* = 1.3 × 10^−5^). In addition, we detected a sharp increase in hypoxanthine upon safranal treatment, which may have a role in oxidative species generation and, ultimately, safranal-induced apoptosis.

**Figure 2:**
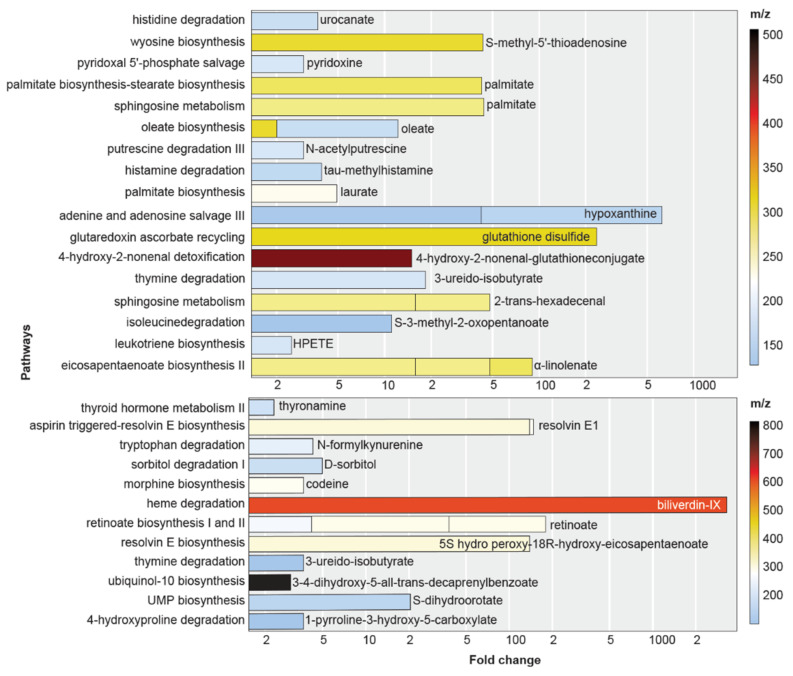
Up (top) and down (bottom)-regulated metabolic pathways in pellet fractions from safranal-treated HCC cells as predicted from the HPLC–MS results in the Systems Biology module of XCMS [17]. These pathways were based on metabolic pathways annotated in the BioCyc4 (biocyc.org) and Uniprot6 (uniprot.org) databases. Their respective metabolites, degree fold-change (*x*-axis), and mass-to-charge ratios (*m*/*z*, heatmap) are shown. Dysregulated metabolites are indicated inside or adjacent to bars for increased (top) or decreased (below) metabolites in safranal-treated cells. Multiple adducts for the same compound are shown as stacked bars.

Metabolites were mapped to known human metabolic pathways using the XCMS Systems Biology module [17,19]. Integrated pathway analysis uses multi-scale database matching and retrieval tools, including KEGG and BioCyc, to predict metabolic pathway dysregulation from MS-detected metabolite clusters (see Figure 2). The results of this semi-automatic annotation algorithm were manually compared with transcript expression values to infer reaction directions.

**Figure 3:**
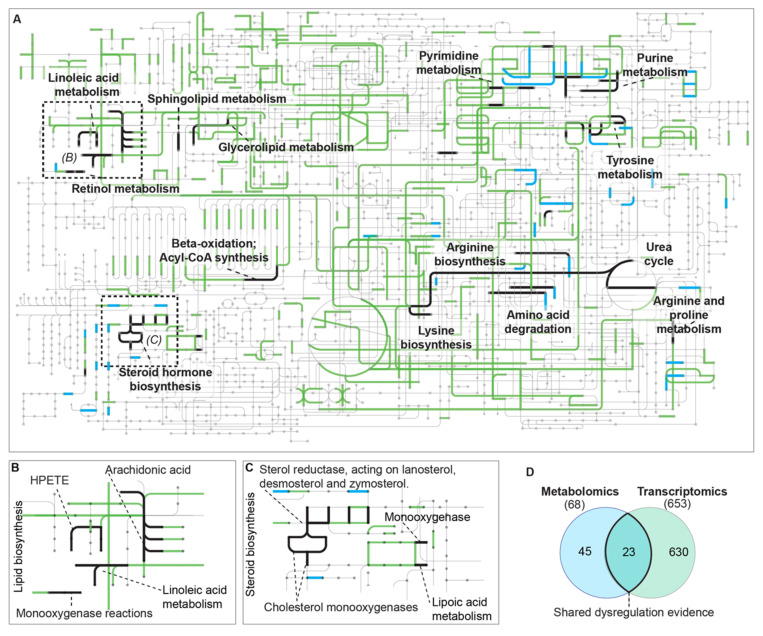
Overlap of metabolomic- and transcriptomic-predicted pathway dysregulation (as observed from cell pellets) in safranal-treated HCC cells. Maps show metabolites (gray dots), edges with no evidence (gray edges), transcriptomic reaction dysregulation evidence (green edges), metabolomic reaction dysregulation evidence (blue edges), and reaction dysregulation evidence from both omics experiments (bold black edges) after safranal treatment. Metabolites and their respective metabolic pathways were identified using LC/MS–QToF and Mummichog (http://mummichog.org/, accessed on 20 March 2021) in XCMS and their overlaps with those identified from RNAseq analysis (black edges, panels A–C). (**A**) Overview of metabolic dysregulation evidence from the dual omics experiments visualized in the Interactive Pathways Explorer v3 (https://pathways.embl.de/, accessed 29 May 2021). (**B**) Lipid biosynthesis dysregulation after safranal treatment (see also Table S2). (**C**) Steroid biosynthesis dysregulation after safranal treatment (see also Table S2). (**D**) Overlap of metabolomics and transcriptomics dysregulated metabolic reactions from CAS and EC numbers. Metabolite accessions were retrieved from the Chemical Abstract Service (CAS) using the METLIN (https://metlin.scripps.edu, accessed 4 May 2021) batch search tool, ECs were retrieved from transcripts using EC Domain Miner [22]. The CAS and EC accession numbers were compared in iPATH3 (https://pathways.embl.de/, accessed 29 May 2021) to detect dysregulated.

We manually extracted Enzyme Commission (EC) codes from differentially expressed and accumulated transcripts and compounds and revealed reactions with overlapping evidence from protein family (PFAM)-derived ECs and Chemical Abstract Service (CAS) registry-derived ECs. The Interactive Pathways Explorer (v3) tool was used to examine overlaps from metabolomic and transcriptomic datasets. Dysregulated metabolites from our HPLC–qTOF–MS studies (*p* < 0.001) were matched against the CAS registry to find associated ECs in Metlin (https://metlin.scripps.edu/, accessed 4 May 2021). The ECs were derived from their PFAM correlates using HMMsearch on translated transcripts, then ECdomainMiner to extract EC numbers from PFAMs with gold-level designations [22]. A total of 653 ECs from DEGs and 68 from CAS accessions in dysregulated metabolites were identified. Twenty-three ECs overlapped in these two datasets (Figure 3). The overlapping ECs corresponded to 47 distinct reactions, including reactions in the urea cycle, fatty acid elongation in mitochondria, arachidonic acid metabolism, pyrimidine metabolism, tyrosine metabolism, and tryptophan metabolism (Figure 3). Metabolic reactions with dual omics evidence for safranal perturbation can be visualized in the iPath metabolic network map in Figure 3 and can also be found in Tables S1 and S2.

### 3.2. Safranal Induces Shifts in the HCC Nucleoside Landscape

We observed a variety of nucleotide derivatives upregulated after safranal treatment. For example, S-methyl-5′-thioadenosine (MTA; CAS: 2457-80-9, KEGG: C00170, PubChem CID: 439176), a sulfur-containing nucleoside, was significantly upregulated after safranal treatment.

Other metabolomic and transcriptomic evidence also implied that the mitochondrial uncoupling process (e.g., futile, thermogenic cycling) was defunct in safranal-treated HCC cells. The generation of ROS is a hallmark of unchecked productive mitochondrial electron flow, and mitochondrial decoupling is a canonical cytoprotective response to the resultant oxidative damage [32,33]. Our data suggest that safranal-treated HCC may not correctly uncouple oxidative phosphorylation and, ultimately, accumulate cytotoxic levels of adenosines. Safranal was recently shown to inhibit the highly conserved ATP synthase [34]; hence, the accumulation of high levels of ATP precursors we observed may be due to mitochondrial electron transport chain (mETC) blockage at the final, productive step.

Hypoxanthine, an ATP derivative (ATP<=>ADP<=>AMP --> adenosine --> inosine<=>hypoxanthine) and guanosine analog were significantly increased after safranal treatment (538-fold increase, *p* = 7.7 × 10^−6^). Hypoxanthine, adenine, and MTA were among the highest-upregulated metabolites (Table S2). Hypoxanthine accumulation was a primary feature of safranal-treated HCC cells; similar increases (up to 600-fold) in hypoxanthine were seen in cells unable to convert inosine monophosphate (IMP) to xanthosine monophosphate (XMP) or AMP and unable to remove dITP/ITP and dXTP/XTP from the nucleotide pool [35]. Hypoxanthine accumulation could result from cytosolic xanthine dehydrogenase (XDH; NSG00000158125) down-regulation, which we observed in the transcriptomic data (3.57-fold decrease, *p* = 0.0015). Hypoxanthine is a prolific free-radical generator that causes apoptosis [36]. We hypothesize that elevated levels of hypoxanthine are likely a strong driver for safranal-induced apoptosis in HCC.

### 3.3. Safranal Induces ROS-Damage Biomarkers and Antioxidant Gene Expression

Several metabolite and transcript dysregulation events coordinately suggested a pro-oxidant environment after safranal treatment. The up-regulation of an antioxidant defense network was outlined in our transcriptomic data, and increases in biomarkers for ROS damage were increased in our metabolomics data. For example, oxidized glutathione was significantly increased after treatment (GSSG; fold-increase = 236.6, *p* = 1.6 × 10^−5^).

The protein KEAP1 was significantly up-regulated (base mean = 1526.5, t = 24.9, *p* = 0). KEAP1 interacts with NRF2, a master regulator of the antioxidant response [37,38,39,40]. The KEAP1 up-regulation reveals an antioxidant response to safranal in HCC cells and suggests that safranal induces a reactive oxygen species (ROS)-rich intracellular environment. Transcripts for the spermidine/spermine acetyltransferase (SAT1; SPD/SPM acetyltransferase), a key enzyme in the polyamine breakdown pathway, significantly decreased (6.1-fold decrease, *p* = 1.65 × 10^−193^). SAT1 acetylates spermidine and modulates polyamine levels by regulating their transport at the cell membrane [41]. The downregulation of SAT1 implies that polyamine processing at the cell membrane is not functioning correctly.

The heme degradation product biliverdin IX [42] decreased after safranal treatment. The biliverdin IX degradation enzyme, biliverdin reductase B (BLVRB, ENSG00000090013, [26]), increased transcript expression after safranal treatment (1.32 fold-increase, *p* = 0.00689). The negative correlation of metabolite and enzyme implies that increased enzymatic activity of BLVRB is responsible for Billiverdin IX decrease in safranal-treated cells. Biliverdin reductase (BVR) was not significantly dysregulated. However, the flavin reductase that reduces biliverdin to bilirubin (BLVRB, NSG00000090013, EC:1.3.1.243) was significantly upregulated (0.4-fold increase, *p* = 0.007). This enzyme also produces NADH [42]. The decrease in biliverdin we observed is likely due to degradation from the upregulated BLVRB in safranal-treated cells.

Intracellular nitric oxide (NO) is also a reactive oxygen species; however, NO precursor reactions were significantly downregulated (Table 1 and Table 2, Figure 3). Metabolites and transcripts for enzymes in the L-arginine biosynthesis pathway were significantly dysregulated (Figure 3). The arginine biosynthetic gene, ARG2, was significantly down-regulated (base mean expression = 90.76 TPM, t = –15.8, *p* = 7.69 × 10^−54^). An inducible NO synthase (NOS2, ENSG00000007171) was downregulated at the transcript level (–2.5 log-fold change; *p* = 3.36 × 10^−6^). NOS2 generates NO, a messenger molecule with diverse functions in multiple human tissues [43,44,45]. Transcription of the arginine synthase ARG2 was also downregulated (Table S1). The downregulation of both ARG2 and NOS2 is likely to decrease arginine and NO fluxes.

The tryptophan degradation pathway via tryptamine appeared to decrease in conditioned media extracts from safranal-treated HCC cells (1.7-fold decrease, *p* = 1.4 × 10^−7^). This result suggests that although safranal-treated HCC cells are apoptotic, and their release of tryptophan degradation products into extracellular space is less. Pathways predicted to be upregulated from media extract evidence included eicosapentaenoate biosynthesis II (3.3-fold increase, *p* = 1.1 × 10^−9^), leukotriene biosynthesis (2-fold increase, *p* = 1.1 × 10^−5^), zymosterol biosynthesis (23.7-fold increase, *p* = 1 × 10^−6^), and retinoate biosynthesis I and II (55.6-fold increase, *p* = 1.9 × 10^−5^). Cell membrane-type lipids increased in the conditioned media extracts, which may be indicative of increased apoptosis or other cell lysis after safranal treatment.

## 4. Discussion

This study used a dual omics workflow to study the phenotypic and transcript expression in HCC to safranal. Pathways involved in the oxidative stress response were predominant features with dysregulation evidence from both omics datasets. Their coordination reveals the apoptosis-inducing effects of safranal through redox instability and shifts in energy homeostasis, ultimately leading to widespread protein destabilization and DNA damage.

The evidence for an activated antioxidant defense in liver cells by a canonical mechanism, coupled with significantly increased oxidized GSSG, hypoxanthine, and various other ROS-damage biomarkers, outlines a highly pro-oxidant, ROS-rich environment induced by safranal. Farahmand et al. suggested that safranal acts as a minor pro-oxidant that activates endogenous antioxidant defenses [46]. The mechanism underlying safranal’s induction of ROS may be based on its inactivation of endogenous antioxidant systems. For example, exogenously applied safranal inhibits catalase in plants [47]. Aside from catalase, the biliverdin–bilirubin cycle is important to maintain redox homeostasis in mammals [48]. When reduced by BLVRB, reactive oxygen species can be formed. BLVRB is a promiscuous enzyme and catalyzes many other pyridine-nucleotide-dependent reductions, including various flavins, biliverdins, quinone, and iron [49]. The reduction of biliverdin by biliverdin reductase A (BLVRA) to bilirubin is a central mechanism for redox regulation in humans; depletion of BLVRA sensitizes human diploid fibroblasts to oxidative stress [50]. Thus, the cycling of biliverdin to bilirubin and back constitutes a redox-modulation circuit that also appears to be perturbed after safranal treatment.

The decrease in the ubiquinol precursor 3-4-dihydroxy-5-all-trans-decaprenylbenzoate (3-fold decrease, *p* = 1.9 × 10^−5^) signifies the loss of an important electron carrier in the mETC. With ubiquinol-10 in the mETC, electrons from upstream mETC components are more likely to result in ROS from electron leak. MTA is a substrate for MTA phosphorylase (EC 2.4.2.28) and produces adenine and S-methyl-5-thio-alpha-D-ribose 1-phosphate [51]. A further downstream product, adenylthiomethylpentose, lowers mammalian body temperature [52]. The increase of MTA suggests that intracellular cryopreservation mechanisms, such as non-shivering thermogenesis mediated by mitochondrial uncoupling, may decrease after safranal treatment.

Nitrogen metabolism appeared to be heavily affected by safranal. Urea, arginase, and nitric oxide (NO) precursor metabolic reactions were significantly dysregulated from transcriptomics and metabolomics evidence. Their disruption of these pathways leads to purine metabolism defects [53,54], in essence, simulating the genetic defects described by Pang et al. The inability to remove excess nucleotides or convert them to downstream products may be responsible for the hypoxanthine accumulation and subsequent oxidative damage in the HCC HEP-G2 cells. The significant increases observed in purine derivatives may be due to interruption of the mETC, which eliminates thermogenesis and promotes the accumulation of excessive ATP or ADP and reactive oxygen species levels due to the resulting electron leaks.

## 5. Conclusions

Overall, protein destabilization that leads to ROS generation appears to be a hallmark of safranal’s mode of action on HCC cells. Save et al. found that safranal caused the inhibition and dis-aggregation of a wide variety of α-synuclein in *E. coli* [23]. This disassociation resulted from safranal binding to the hydrophobic parts of proteins, thus interfering with protein folding. Ultimately, interference in intracellular protein folding has a cytotoxic effect, consistent with our previous conclusions [5] and the results in this study. One of the key features we observed that may stem from protein destabilization was the accumulation of hypoxanthine. In itself, hypoxanthine has been shown to induce apoptosis through an ROS-dependant mechanism by the induction of oxidative stress in endothelial cells [36]. Thus, protein destabilization leading to hypoxanthine accumulation appears to be the primary mechanism of cytoxicity from safranal treatment of HCC cells.

## Figures and Tables

**Table 1:** Metabolites significantly upregulated after 24 h safranal treatment in pellet extracts involved in critical metabolic pathways.

Metabolites	Pathways Involved	Fold- Change	*p*-Value	*m*/*z*	Retention Time	Adduct
**α-linolenate**	eicosapentaenoate biosynthesis II	32.2	2.2 × 10^−7^	261.2201	1.4	M-H_2_O[1+]
**α-linolenate**	eicosapentaenoate biosynthesis II	42.5	2 × 10^−6^	279.2305	1.39	M+H[1+]
**HPETE**	leukotriene biosynthesis	2.5	6 × 10^−6^	169.1215	0.85	M+2H[2+]
**S-3-methyl-2-** **oxopentanoate**	isoleucine degradation	11.1	4.4 × 10^−7^	113.0591	0.75	M-H_2_O[1+]
**2-trans-hexadecenal**	sphingosine metabolism	15.8	1.2 × 10^−5^	261.2201	0.87	M+Na[1+]
**2-trans-hexadecenal**	sphingosine metabolism	32.2	2.2 × 10^−7^	261.2201	1.4	M+Na[1+]
**3-ureido-isobutyrate**	Thymine degradation	18.4	3.6 × 10^−5^	169.0579	1.06	M+Na[1+]
**4-hydroxy-2-nonenal-glutathione conjugate**	4-hydroxy-2-nonenal detoxification	15	1.2 × 10^−6^	465.2138	5.09	M+H[1+]
**glutathione disulfide**	glutaredoxin ascorbate recycling	236.6	1.6 × 10^−5^	308.0897	0.52	M+2H[2+]
**hypoxanthine**	purine degradation	42.3	7.7 × 10^−5^	119.0346	0.52	M-H_2_O[1+]
**hypoxanthine**	purine degradation	583.7	7.7 × 10^−6^	137.0451	0.53	M+H[1+]
**laurate**	palmitate biosynthesis	4.9	3 × 10^−5^	223.1667	0.79	M+Na[1+]
**methylhistamine**	histamine degradation	3.9	3.4 × 10^−7^	144.1374	0.77	M+NH_3_[1+]
**N-acetylputrescine**	putrescine degradation III	3	1.8 × 10^−6^	170.081	0.73	M+K[1+]
**oleate**	oleate biosynthesis	2	5.5 × 10^−5^	300.2884	3.19	M+NH_3_[1+]
**oleate**	oleate biosynthesis	10.2	4 × 10^−5^	153.1264	1.37	M+H+Na
**palmitate**	aldehydesphingosine andsphingosine-1-phosphate metabolism	43.8	8 × 10^−7^	263.2354	1.34	M+Na[1+]
**palmitate**	palmitate biosynthesis-stearate biosynthesis	42.5	2 × 10^−6^	279.2305	1.39	M+Na[1+]
**pyridoxine**	pyridoxal 5′-phosphate salvage	3	1.8 × 10^−6^	170.081	0.73	M+H[1+]
**S-methyl-5′-** **thioadenosine**	wyosine biosynthesis	43.4	7.2 × 10^−5^	298.0963	0.54	M+H[1+]
**urocanate**	histidine degradation	3.7	2 × 10^−7^	156.076	0.5	M+NH3+

**Table 2:** Metabolites significantly downregulated after 24 h safranal treatment in media extracts involved in critical metabolic pathways.

Metabolites	Pathways Involved	Fold- Change	*p*-Value	*m*/*z*	Retention Time	Adduct
**1-pyrroline-3-hydroxy-5-** **carboxylate**	4-hydroxyproline degradation	–3.7	1.3 × 10^−5^	130.0493	0.5	M+H[1+]
**S-dihydroorotate**	UMP biosynthesis	–20.5	1.8 × 10^−5^	176.066	1.21	M+NH_3_[1+]
**3-4-dihydroxy-5-all-trans-** **decaprenylbenzoate**	ubiquinol-10 biosynthesis	–3	1.9 × 10^−5^	835.659	8.84	M+H[1+]
**3-ureido-isobutyrate**	thymine degradation	–3.7	1.3 × 10^−5^	130.0493	0.5	M-NH_3_[1+]
**5S hydro peroxy-18R-hydroxy-eicosapentaenoate**	resolvin E biosynthesis	–138.8	4 × 10^−5^	333.2044	1.57	M-H_2_O[1+]
**all-trans-retinoate**	retinoate biosynthesis	–4.2	2.5 × 10^−8^	283.2043	2.22	M-H_2_O[1+]
**all-trans-retinoate**	retinoate biosynthesis	–33.9	1.2 × 10^−5^	323.1968	1.11	M+Na[1+]
**all-trans-retinoate**	retinoate biosynthesis	–141.3	4.2 × 10^−6^	323.1966	1.53	M+Na[1+]
**biliverdin-IX-**	α-heme degradation	–3281.8	5.3 × 10^−6^	600.2797	1.28	M+NH_3_[1+]
**codeine**	morphine biosynthesis	–3.7	8.2 × 10^−5^	318.1929	0.84	M+NH_3_[1+]
**D-sorbitol**	sorbitol degradation I	–5	3.1 × 10^−5^	205.0675	1.16	M+Na[1+]
**N-formylkynurenine**	tryptophan degradation	–4.3	5 × 10^−5^	255.1223	0.86	M+NH_3_[1+]
**resolvin E1**	resolvin E biosynthesis	–138.8	4 × 10^−5^	333.2044	1.57	M-H_2_O[1+]
**resolvin E2**	resolvin E biosynthesis	–8.8	2.6 × 10^−5^	317.2101	0.88	M-H_2_O[1+]

## Data Availability

All Supplementary Data are hosted at Zenodo (zenodo.org (doi:10.5281/zenodo.4299903, accessed on 29 April 2022). Sequencing reads are hosted at the National Center for Biotechnology Information (NCBI) sequence read archive (SRA) with the BioProject accession PRJNA682690.

## Supplementary Materials

The following supporting information can be downloaded at: https://www.mdpi.com/article/10.3390/antiox11061125/s1, The following are available online: Table S1: Differentially-expressed genes and their predicted EC accessions, Table S2: tabular summary of XCMS suite results for triplicates of each control and treated cells, including *m*/*z*, fold-change, and retention times. Data S1: Hosted at zenodo.org (doi:10.5281/zenodo.4299903, accessed on 29 April 2022). RNAseq data and analyses (tar.gz archive), Data S2: Metabolomic Data and Analyses (.zip archive).

## References

[1] Jindal A., Thadi A., Shailubhai K. (2019). Hepatocellular Carcinoma: Etiology and Current and Future Drugs. J. Clin. Exp. Hepatol..

[2] Zhu R.X., Seto W.-K., Lai C.-L., Yuen M.-F. (2016). Epidemiology of Hepatocellular Carcinoma in the Asia-Pacific Region. Gut Liver.

[3] Liu Y.C., Chen K.-F., Chen P.-J. (2015). Treatment of liver cancer. Cold Spring Harb. Perspect. Med..

[4] Yao Z., Bhandari A., Wang Y., Pan Y., Yang F., Chen R., Xia E., Wang O. (2018). Dihydroartemisinin potentiates antitumor activity of 5-fluorouracil against a resistant colorectal cancer cell line. Biochem. Biophys. Res. Commun..

[5] Tarantilis P.A., Polissiou M., Manfait M. (1994). Separation of picrocrocin, cis-trans-crocins and safranal of saffron using high-performance liquid chromatography with photodiode-array detection. J. Chromatogr. A.

[6] Hoshyar R., Mollaei H. (2017). A comprehensive review on anticancer mechanisms of the main carotenoid of saffron, crocin. J. Pharm. Pharmacol..

[7] Bathaie S.Z., Bolhassani A., Tamanoi F. (2014). Anticancer Effect and Molecular Targets of Saffron Carotenoids. Enzymes.

[8] Bajbouj K., Schulze-Luehrmann J., Diermeier S., Amin A., Schneider-Stock R. (2012). The anticancer effect of saffron in two p53 isogenic colorectal cancer cell lines. BMC Complement. Altern. Med..

[9] Amin A., Hamza A.A., Bajbouj K., Ashraf S.S., Daoud S. (2011). Saffron: A potential candidate for a novel anticancer drug against hepatocellular carcinoma. Hepatology.

[10] Samarghandian S., Boskabady M.H., Davoodi S. (2010). Use of in vitro assays to assess the potential antiproliferative and cytotoxic effects of saffron (Crocus sativus L.) in human lung cancer cell line. Pharmacogn. Mag..

[11] Farahzad J.A., Samarghandian S., Shoshtari M.E., Sargolzaei J., Hossinimoghadam H. (2014). Anti-tumor activity of safranal against neuroblastoma cells. Pharmacogn. Mag..

[12] Al-Hrout A., Chaiboonchoe A., Khraiwesh B., Murali C., Baig B., El-Awady R., Tarazi H., Alzahmi A., Nelson D.R., Greish Y.E. (2018). Safranal induces DNA double-strand breakage and ER-stress-mediated cell death in hepatocellular carcinoma cells. Sci. Rep..

[13] Nelson D.R., Chaiboonchoe A., Fu W., Hazzouri K.M., Huang Z., Jaiswal A.K., Daakour S., Mystikou A., Arnoux M., Sultana M. (2019). Potential for Heightened Sulfur-Metabolic Capacity in Coastal Subtropical Microalgae. iScience.

[14] Castro-Perez J.M., Kamphorst J., DeGroot J., Lafeber F., Goshawk J., Yu K., Shockcor J.P., Vreeken R.J., Hankemeier T. (2010). Comprehensive LC−MS ^E^ Lipidomic Analysis using a Shotgun Approach and Its Application to Biomarker Detection and Identification in Osteoarthritis Patients. J. Proteome Res..

[15] Smith C.A., Want E.J., O’Maille G., Abagyan R., Siuzdak G. (2006). XCMS: Processing Mass Spectrometry Data for Metabolite Profiling Using Nonlinear Peak Alignment, Matching, and Identification. Anal. Chem..

[16] Liu Q., Shi Y., Guo T., Wang Y., Cong W., Zhu J. (2012). Metabolite discovery of helicidum in rat urine with XCMS based on the data of ultra performance liquid chromatography coupled to time-of-flight mass spectrometry. J. Chromatogr. B Anal. Technol. Biomed. Life Sci..

[17] Huan T., Forsberg E.M., Rinehart D., Johnson C.H., Ivanisevic J., Benton H.P., Fang M., Aisporna A., Hilmers B., Poole F.L. (2017). Systems biology guided by XCMS Online metabolomics. Nat. Methods.

[18] Gowda H., Ivanisevic J., Johnson C.H., Kurczy M.E., Benton H.P., Rinehart D., Nguyen T., Ray J., Kuehl J., Arevalo B. (2014). Interactive XCMS Online: Simplifying Advanced Metabolomic Data Processing and Subsequent Statistical Analyses. Anal. Chem..

[19] Forsberg E.M., Huan T., Rinehart D., Benton H.P., Warth B., Hilmers B., Siuzdak G. (2018). Data processing, multi-omic pathway mapping, and metabolite activity analysis using XCMS Online. Nat. Protoc..

[20] Love M.I., Huber W., Anders S. (2014). Moderated estimation of fold change and dispersion for RNA-seq data with DESeq. Genome Biol..

[21] Krämer A., Green J., Pollard J., Tugendreich S. (2014). Causal analysis approaches in Ingenuity Pathway Analysis. Bioinformatics.

[22] Alborzi S.Z., Devignes M.-D., Ritchie D.W. (2017). ECDomainMiner: Discovering hidden associations between enzyme commission numbers and Pfam domains. BMC Bioinform..

[23] Save S.S., Rachineni K., Hosur R.V., Choudhary S. (2019). Natural compound safranal driven inhibition and dis-aggregation of α-synuclein fibrils. Int. J. Biol. Macromol..

[24] Stewart G.R., Robertson B.D., Young D.B. (2004). Analysis of the function of mycobacterial DnaJ proteins by overexpression and microarray profiling. Tuberculosis.

[25] Shu B., Jia J., Zhang J., Sethuraman V., Yi X., Zhong G. (2018). DnaJ homolog subfamily A member1 (DnaJ1) is a newly discovered anti-apoptotic protein regulated by azadirachtin in Sf9 cells. BMC Genom..

[26] Stark J.L., Mehla K., Chaika N., Acton T.B., Xiao R., Singh P.K., Montelione G.T., Powers R. (2014). Structure and Function of Human DnaJ Homologue Subfamily A Member 1 (DNAJA1) and Its Relationship to Pancreatic Cancer. Biochemistry.

[27] Stark J., Mercier K.A., Mueller G., Acton T.B., Xiao R., Montelione G.T., Powers R. (2010). Solution structure and function of YndB, an AHSA1 protein from Bacillus subtilis. Proteins.

[28] Sheehan-Rooney K., Swartz M.E., Zhao F., Liu D., Eberhart J.K. (2013). Ahsa1 and Hsp90 activity confers more severe craniofacial phenotypes in a zebrafish model of hypopar-athyroidism, sensorineural deafness and renal dysplasia (HDR). Dis. Models Mech..

[29] Shao J., Wang L., Zhong C., Qi R., Li Y. (2016). AHSA1 regulates proliferation, apoptosis, migration, and invasion of osteosarcoma. Biomed. Pharmacother..

[30] Woodford M.R., Sager R.A., Marris E., Dunn D.M., Blanden A.R., Murphy R.L., Rensing N., Shapiro O., Panaretou B., Prodromou C. (2017). Tumor suppressor Tsc1 is a new Hsp90 co-chaperone that facilitates folding of kinase and non-kinase clients. EMBO J..

[31] Cheriyamundath S., Choudhary S., Lopus M. (2018). Safranal Inhibits HeLa Cell Viability by Perturbing the Reassembly Potential of Microtubules. Phytotherapy Res..

[32] Cadenas S., Aragonés J., Landázuri M.O. (2010). Mitochondrial reprogramming through cardiac oxygen sensors in ischaemic heart disease. Cardiovasc. Res..

[33] Garcia J., Han D., Sancheti H., Yap L.-P., Kaplowitz N., Cadenas E. (2010). Regulation of Mitochondrial Glutathione Redox Status and Protein Glutathionylation by Respiratory Substrates. J. Biol. Chem..

[34] Liu M., Amini A., Ahmad Z. (2017). Safranal and its analogs inhibit Escherichia coli ATP synthase and cell growth. Int. J. Biol. Macromol..

[35] Pang B., McFaline J.L., Burgis N.E., Dong M., Taghizadeh K., Sullivan M.R., Elmquist C.E., Cunningham R.P., Dedon P.C. (2012). Defects in purine nucleotide metabolism lead to substantial incorporation of xanthine and hypoxanthine into DNA and RNA. Proc. Natl. Acad. Sci. USA.

[36] Kim Y.J., Ryu H.M., Choi J.Y., Cho J.H., Kim C.D., Park S.H., Kim Y.L. (2017). Hypoxanthine causes endothelial dysfunction through oxidative stress-induced apoptosis. Biochem. Biophys. Res. Commun..

[37] Clerici S., Boletta A. (2020). Role of the KEAP1-NRF2 Axis in Renal Cell Carcinoma. Cancers.

[38] Fakhri S., Pesce M., Patruno A., Moradi S.Z., Iranpanah A., Farzaei M.H., Sobarzo-Sánchez E. (2020). Attenuation of Nrf2/Keap1/ARE in Alzheimer’s Disease by Plant Secondary Metabolites: A Mechanistic Review. Molecules.

[39] Moretti D., Tambone S., Cerretani M., Fezzardi P., Missineo A., Sherman L.T., Munoz-Sajuan I., Harper S., Dominquez C., Pacifici R. (2020). NRF2 activation by reversible KEAP1 binding induces the antioxidant response in primary neurons and astrocytes of a Huntington’s disease mouse model. Free Radic. Biol. Med..

[40] Thanas C., Ziros P.G., Chartoumpekis D.V., Renaud C.O., Sykiotis G.P. (2020). The Keap1/Nrf2 Signaling Pathway in the Thyroid-2020 Update. Antioxidants.

[41] Mandal S., Mandal A., Park M.H. (2015). Depletion of the polyamines spermidine and spermine by overexpression of spermi-dine/spermine N(1)-acetyltransferase 1 (SAT1) leads to mitochondria-mediated apoptosis in mammalian cells. Biochem. J..

[42] Yamaguchi T., Komoda Y., Nakajima H. (1994). Biliverdin-IX alpha reductase and biliverdin-IX beta reductase from human liver. Purification and characterization. J. Biol. Chem..

[43] Nikolaidis A., Kramer R., Ostojic S. (2021). Nitric Oxide: The Missing Factor in COVID-19 Severity?. Med. Sci..

[44] Peluffo R.D. (2021). Cationic amino acid transporters and their modulation by nitric oxide in cardiac muscle cells. Biophys. Rev..

[45] Hashemi M., Karami M., Zarrindast M.R. (2022). The regulatory role of nitric oxide in morphine-induced analgesia in the descending path of pain from the dorsal hippocampus to the dorsolateral periaqueductal gray. Eur. J. Pain.

[46] Farahmand S.K., Samini F., Samini M., Samarghandian S. (2013). Safranal ameliorates antioxidant enzymes and suppresses lipid peroxidation and nitric oxide formation in aged male rat liver. Biogerontology.

[47] Mardani H., Maninang J., Appiah K.S., Oikawa Y., Azizi M., Fujii Y. (2019). Evaluation of Biological Response of Lettuce (Lactuca sativa L.) and Weeds to Safranal Allelochemical of Saffron (Crocus sativus) by Using Static Exposure Method. Molecules.

[48] Barañano D.E., Rao M., Ferris C.D., Snyder S.H. (2002). Biliverdin reductase: A major physiologic cytoprotectant. Proc. Natl. Acad. Sci. USA.

[49] Wang J., de Montellano P.R.O. (2003). The binding sites on human heme oxygenase-1 for cytochrome p450 reductase and biliverdin reductase. J. Biol. Chem..

[50] Kim S.Y., Kang H.T., Choi H.R., Park S.C. (2011). Biliverdin reductase A in the prevention of cellular senescence against oxidative stress. Exp. Mol. Med..

[51] Kamatani N., Nelson-Rees W.A., Carson D.A. (1981). Selective killing of human malignant cell lines deficient in methylthioadenosine phosphorylase, a purine metabolic enzyme. Proc. Natl. Acad. Sci. USA.

[52] Kuhn R., Henkel K. (1941). Über die Senkung der Körpertemperatur durch Adenylthiomethylpentose. Hoppe Seyler’s Z. Physiol. Chemie.

[53] Lindberg J.E., Jacobsson K.-G. (1990). Nitrogen and purine metabolism at varying energy and protein supplies in sheep sustained on intragastric infusion. Br. J. Nutr..

[54] Werner L., Dreyer J.H., Hartmann D., Barros M.H.M., Büttner-Herold M., Grittner U., Niedobitek G. (2020). Tumor-associated macrophages in classical Hodgkin lymphoma: Hormetic relationship to outcome. Sci. Rep..

